# A Genome-Wide Survey of Genetic Instability by Transposition in Drosophila Hybrids

**DOI:** 10.1371/journal.pone.0088992

**Published:** 2014-02-20

**Authors:** Doris Vela, Antonio Fontdevila, Cristina Vieira, María Pilar García Guerreiro

**Affiliations:** 1 Grup de Biología Evolutiva, Departament de Genètica i Microbiologia, Facultat de Biociències, Universitat Autònoma de Barcelona, Bellaterra, Barcelona, Spain; 2 Laboratoire de Biométrie et Biologie Evolutive, UMR5558, Université Lyon1, Villeurbanne, France; 3 Institut Universitaire de France, Paris, France; Virginia Tech Virginia, United States of America

## Abstract

Hybridization between species is a genomic instability factor involved in increasing mutation rate and new chromosomal rearrangements. Evidence of a relationship between interspecific hybridization and transposable element mobilization has been reported in different organisms, but most studies are usually performed with particular TEs and do not discuss the real effect of hybridization on the whole genome. We have therefore studied whole genome instability of Drosophila interspecific hybrids, looking for the presence of new AFLP markers in hybrids. A high percentage (27–90%) of the instability markers detected corresponds to TEs belonging to classes I and II. Moreover, three transposable elements (*Osvaldo, Helena and Galileo*) representative of different families, showed an overall increase of transposition rate in hybrids compared to parental species. This research confirms the hypothesis that hybridization induces genomic instability by transposition bursts and suggests that genomic stress by transposition could contribute to a relaxation of mechanisms controlling TEs in the Drosophila genome.

## Introduction

Natural hybridization is a well known phenomenon in organisms living in sympatry and constitutes an important mechanism of speciation [1], [2]. Whereas a large number of natural hybridization examples were reported in plants [3], [4], natural hybrid reports in animals are less frequent likely due to the lack of suitable markers for their detection. The importance of hybridization in evolution of species has been debated for decades and is under major reevaluation [1], [2], [5], [6]. One of the most evaluated consequences of hybridization is that the merging of two different genomes triggers a “genomic shock” leading to genomic modifications including cascades of new gene expressions often accompanied by transposable element (TE) mobilizations [7]. TEs are found in genomes of almost all living organisms [8]. Considered as enigmatic sequences with an uncertain role in the genome, we now know their importance in the building of the genome [9] and in particular its regulation [9]. Their mobility in the genome is usually regulated at a low transposition frequencies, which may greatly increase when different stresses deregulate them [10]. Barbara McClintock in the early 1980s proposed that TEs could be activated by a genomic shock, like hybridization, which could confer an adaptive value to the host. The role of TEs has been ascribed to hybrid incompatibility and speciation [11], [12], intraspecific crosses between different Drosophila strains can trigger the mobilization of different TEs. The best known examples are hybrid dysgenesis mechanisms P-M [13], [14], I-R [15], H-E [16] in *D. melanogaster* and the dysgenesis of *Penelope* and other elements in *D. virilis* [17]. Different elements are sometimes mobilized simultaneously [17] due to an absence of maternal piRNAs impeding the mobilization of paternally inherited families in dysgenic crosses [18] and *D. simulans* intraspecific crosses involving the retrotransposon *tirant* [19]. As in Drosophila, most of the organisms have activated mechanisms of epigenetic control to avoid the negative effect produced by high levels of transposition created by genomic stress. During interspecific crosses TE seem to escape to the genomic control allowing their mobilization and the creation of new insertions. Genomic stresses due to hybridization have an impact on TE mobilization and activity in plants, mammals and insects [20]. The early more numerous examples of increase of TE activity in interspecific hybrids have been reported in plants [21]–[24]. For example, in interspecific hybrids of *Solanum lycopersicum* and *Solanum pennellii* epigenetic and gene expression changes due to accumulation of transgressive small RNAs in the hybrid genomes were found [25]. In Drosophila, interspecific hybrids have given sometimes different results; while interspecific hybrids between species from the *melanogaster* subgroup [26] and *affinis* [27] subgroup do not have an increased transposition rate, two well described examples of TEs activation associated to interspecific hybridization are known. The first one concerns kangaroos, where an induction of TE transposition and centromeric expansion was observed in hybrids of wallabies [28], [29]. The second example was found in *Drosophila* where *D. buzzatii* and *D. koepferae* hybrids underwent an increase of transposition of the *Osvaldo* retrotransposon of one order of magnitude higher than in the parental species [30], [31]. The scarcity of animal data is probably due to the difficulty of obtaining a large number of hybrid individuals to perform experimental crosses, technical difficulties in transposition detection, or simply because the direct detection of transposition events is difficult.

Previous work on the same species were restricted to the behaviour of a single element [30], [31]. We ignore whether, in response to genomic stresses, TE mobilization is a universal response for all genomic TEs or, on the contrary, it depends on the kind of element. If this were the case, it would explain why no TE mobilization was found in other Drosophila species. We undertook a genome-wide dissection of TE mobilization in Drosophila interspecific hybrids, including a quantitative estimation of transposition rates of some TEs. Knowledge of the impact of hybridization in TE activation is a prerequisite for understanding the implication of TEs in reproductive isolation, speciation and, eventually, in the evolution of hybrid lineages.

## Materials and Methods

### Drosophila Stocks

*D. buzzatii* Bu28 stock was originated by the fusion of 4 stocks (LN13, 19, 31 and 33) collected in Bolivia (Los Negros) and *D. koepferae* Ko2 stock collected in Argentina (San Luis Valley). Both stocks correspond to inbred lines maintained by brother–sister matings for several years and kept thereafter by mass culturing.

### Crosses

**Figure 1:**
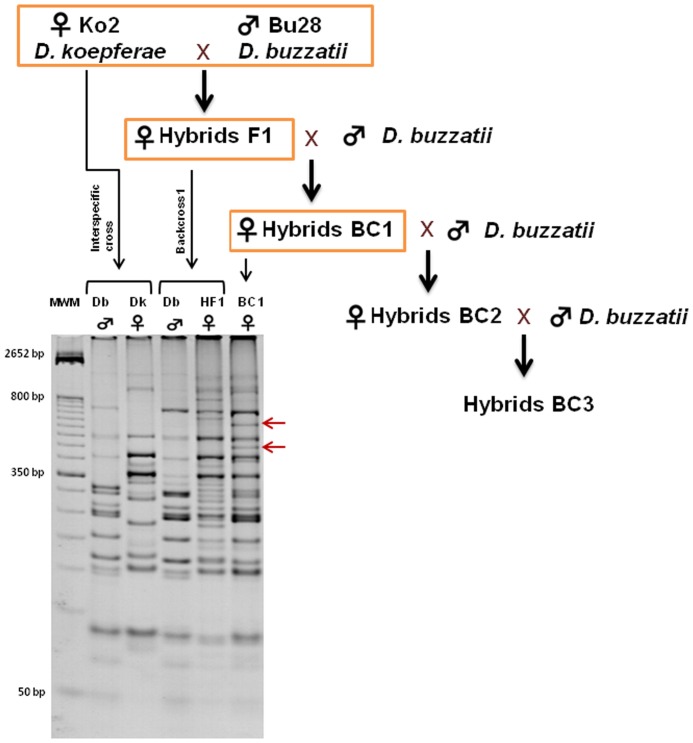
Hybrid crosses and an example of AFLP gel showing bands of parental species and hybrids. **A)** Interspecific cross and backcrosses used in experiments **B)** Selective PCR AFLP band patterns using primers with selective nucleotides GG (*Eco*RI) and CTG (*Mse*I). The arrows indicate two instability markers detected in hybrids from backcross 1 as an example. MWM, molecular weight marker; Dk, *D. koepferae*; Db, *D. buzzatii*; HF1, F1 hybrids; BC1, backcross 1.

50 interspecific crosses (Figure 1) were established between 2 *D. koepferae* females and one *D. buzzatii* male. While the backcross 1 (BC1) was carried out by mass crossing, backcrosses 2 (BC2) and 3 (BC3) were done by individual crosses between a fertile hybrid female and a *D. buzzatii* male. Due to the presence of high number of sterile or semi-sterile females, especially in the F1, we only obtained the complete progeny (from F1 to BC3) of 3 crosses. Families named 10, 13 and 40, were analyzed by AFLP and transposon display and one additional family (family 1) was added to the transposon display experiments (see transposon display section). A segmental hybrid stock F3–F4 was also analyzed by AFLP; these segmental hybrids carry the F3–F4 region of chromosome 4 from *D. koepferae* introgressed in the genomic background of *D. buzzatii.* This cytological region was selected for 60 generations by cytological observations of polytene chromosomes. Simultaneously with the first hybrid crosses, individual intraspecific crosses of *D. buzzatii* and *D. koepferae* parental species were also established and kept for 4 generations as controls in experiments on AFLP (families B4 and K9 of *D. buzzatii* and *D. koepferae* respectively) and transposon display (families B4, B8 of *D. buzzatii* and K3, K9 of *D. koepferae*).

### DNA Extraction and AFLPs

Individual DNA extractions from adult hybrids and individuals from the parental species involved in each cross,stored at −80°C, were performed as described in the Piñol protocol [32]. AFLP markers were obtained using the Vela protocol [33] where the DNA of each individual was digested by a frequent cutter enzyme (*Mse*I) and an infrequent one (*Eco*RI) and then ligated to oligonucleotide adapters. Fragments, after linking an adapter to both extremities, are amplified with primers having a supplementary base. A second round of amplification was given with primers where 3 (for *Mse*I) and 2 nucleotides (*Eco*RI) were added to the 3′ end of the initial primer sequence. The resulting bands were seen on 8% poly-acrylamide gels each containing individual samples from: parental species, parents and the hybrids at the generation being examined (Figure 1). To identify instability, each band present in hybrids was carefully checked for its presence in parents and parental species. Those in hybrids (backcrossed introgression individuals) but absent in parents, parental species and in hybrids of previous generations, were considered new AFLP bands (instability markers) of new genomic rearrangements induced by hybridization that were not exclusively associated to TEs. These selected instability markers were subsequently cloned, sequenced and analyzed. The AFLP technique was chosen because does not need prior knowledge of TEs sequence in species (not yet sequenced) under examination allowing a genome-wide screening from a random sampling of the whole genome.

### Transposon Display

Transposon display is an AFLP-based specific technique [34], [35] allowing the simultaneous amplification of the TE insertions from a particular element. Individual transposons are identified by a ligation-mediated nested PCR that starts within the transposon and amplifies part of the flanking sequence up to a specific restriction site. The resulting PCR products are sequenced and the bands separated according to size. From the putative TE related sequences detected by AFLP as new bands, we have studied only those whose sequence known in our species (not yet sequenced) because this technique requires a previous knowledge of the TE sequence.

We estimated the transposition rates of *Osvaldo*, *Helena* and *Galileo,* three elements belonging to different TE classes and being well characterized in *D. buzzatii*. DNA, from parents and hybrids of each family cross and generation was individually digested with *Hpa*II enzyme (*Osvaldo* and *Galileo* elements) and *Mse*I (*Helena* element), in a total volume of 20 µl (10×enzyme buffer, 1U enzyme, 12 µl H_2_O and 5 µl of DNA) and incubated one hour at 37°C. A mix of adaptors was prepared with 10 µl of each adaptor (10 µM) and 80 µl H_2_O. The adaptors used for *Hpa*II were: 5′AACAGCTGGACGATGAGTCCTGAGATACG 3′ and 5′**CG**CGTATCTCAGGAGTGTA 3′ and for *Mse*I: 5′AAAAGCTGGACGATGAGTCCTGAGA 3′ and 5′**TA**TCTCAGGAGTGTA 3′. The ligation reaction was done in a final volume of 42 µl, including 5×ligase buffer, 2.5 U of T4 DNA ligase, 0.2 µM adaptors mix, 10.7 µl of H_2_O and 20 µl of digestion product, and was later incubated for 1h at 24°C. External amplification was carried out in a final volume of 25 µl including: 1×PCR buffer, 0.2 µM of dNTPs, 0.4 µM *Mse*I primer, 2 µM external primer, 0.625 U DNA polymerase, 17.9 µl H_2_O and 2 µl digestion-ligation product. External amplification was programmed as follows: 2 min 94°C, 25 cycles of [1 min 94°C, 1 min 58°C, 1 min 72°C], 4 min 72°C. The product of external amplification was diluted 20 times with water. The external primers used were: *Osvaldo* 5′AGCCCATTTGCTGACACTTTA3′, *Helena* 5′TGTTGTTGTCATGGTGCTGA3′, and *Galileo* 5′CATGGGGCAGAAAGAGAAAG3′. For the internal amplification, 1.5 µl of the external amplification product (diluted 20 times) was used as template by mixing 10×PCR Buffer, 0.2 µM dNTPs, 0.2 µM primer *Mse*I, 0.25 µM internal primer, 1.25 U DNA polymerase and 15.1 µl of H_2_O. The PCR thermocycler was programmed as follows: 1 min 94°C, 35 cycles of [45 s 94°C, 45 s 58°C, 45 s 72°C], 3 min 72°C. In this last amplification step, specific labelled primers (4, 7, 2′, 4′, 5′, 7′-hexachloro-6-carboxyfluorescein (HEX) fluorochrome) were used: *Osvaldo* 5′CTCTCTGACCCTTCCAGTCG3′, *Helena* 5′GAATTCAGCCCTCAGCTCAA3′, and *Galileo* 5′TTTGGAAAATCGACCGTCAC3′.

Transposon display results were analyzed by capillary electrophoresis, to obtain fragment sizes by Biofidal sequencing services (Lyon, France), and analyzed using the Peak Scanner v4.0 software (Applied Biosystem, CA). Because one enzyme restriction site is inside the element and the other in the flanking sequence, the bands represent the number of insertions. Comparisons between band sizes in hybrid, parents and parental species allowed us to identify those present in hybrids and absent in parents and/or parental species (new bands), which correspond to transposition events.

To verify the reliability of the transposon display technique, several bands per element were sequenced showing that most of them corresponded to the TEs insertions being examinated.

### FISH (Fluorescent *in situ* Hybridization)

Polytene chromosome squashes from salivary glands of third-instar larvae were hybridized with fluorescein labelled probes of *Osvaldo, Helena* and *Galileo*. The *Osvaldo* probe consisted of a 6.4-kb *Osvaldo* fragment [36], and those of *Helena* and *Galileo* consisted of PCR fragments of 440 and 1150 pb respectively containing endonuclease and transposase regions. Prehybridization solutions and posthybridization washes, were done following a protocol by Roche. PCR reactions were carried out in a final volume of 25 µl, including 1× activity buffer (Ecogen), 1.6 mM MgCl2, 0.2 mM of each dNTP (Roche), 0.4 µM primer (SigmaAldrich), 10–20 ng genomic template DNA, and 0.04 units per µl of Taq polymerase (Kapataq from Cultek). Amplifications were run in a MJ Research Inc. thermocycler programmed as follows: 5 min preliminary denaturation at 94°, 30 cycles of 45 s at 94° (denaturation), 45 s at specific PCR annealing temperatures, 1.5 min at 72° (extension) and a final extension for 10 min at 72°. PCR products were gel purified with NucleoSpin kit (Machery-Nagel) and labelled by PCR using Alexa Fluor 488 signal amplification kit (Roche). After hybridization, signal development was done with 2 antibodies: rabbit anti-fluorescein and goat anti-rabbit. Chromosomes were stained with 4′, 6-diamidino-2-phenylindole (DAPI) containing Vectashield mounting media and images taken with a fluorescence microscope.

### Transposition Rates

Transposition rates (T) of *Osvaldo*, *Helena* and *Galileo* were estimated at each generation as: T = Ni/N.2.A [30] where Ni is the number of new insertions (those found in progeny, but not in parents or in the parental stock they come from), N the number of analyzed individuals, 2 being the number of parental genomes and A the number of original insertions (sum of parental insertions). The number of new and original insertions was computed from the copies amplified by transposon display for every TE and is provided as supplementary material (Tables S1). The transposition rate was estimated for 3 hybrid generations (BC1, BC2 and BC3) of 4 hybrid families (1, 10, 13, 40), and for the control families (B4, B8, K3, K9) of parental species at the fourth generation (F4). The transposon display technique cannot distinguish completely between transposition events in somatic and germinal line although if a new band is detected in multiple individuals from the same cross, it probably occurred in the parent germline. In contrast, if a new insertion event is present in only one individual of the progeny, it is impossible to distinguish between transpositions occurring in somatic cells or meiotic germline events.

### RT-PCR

Males of *D. buzzatii* and females of *D. koepferae* parental species were dissected and their testes and ovaries extracted, respectively, in order to separate somatic (carcasses) and germinal (gonads) tissues, and to analyze the transcriptional activity of TEs in each tissue. For this analysis, 10 samples of each tissue were stored at −70°C in a buffer containing beta-mercaptoethanol and RLT buffer (Qiagen) to avoid RNA degradation. RNA extractions were done with RNeasy mini kit (Qiagen) according to the manufacturer’s protocol and treated with DNase I (Ambion) to eliminate DNA contamination. cDNA was synthesized using the Transcriptor First Strand cDNA Syntesis kit (Roche). RT-PCR reactions were carried out in a final volume of 25 µl including 10×PCR buffer, 0.8 mM MgCl_2_, 0.2 µM dNTPs, 0.8 µM primer 1, 0.8 µM primer 2, 1.2 U DNA polymerase, 10 pg cDNA and 13.9 µl H_2_O. Amplifications were run in a thermocycler programmed as follows: 5 min 94°C; 30 cycles of 30 sec at 94°C, 45 sec at 59°C and 30 sec at 72°C; and a final extension of 10 min at 72°C. Reverse transcriptase, endonuclease and transposase regions of *Osvaldo*, *Helena* and *Galileo* respectively, were amplified using the following RT-PCR primers: 5′GAGGCACGAACTGGAGAAAT3′ and 5′ACTCCCATTTGACGCCCTTT3′ for *Osvaldo,* 5′CGACATACTCGCTTCCTGTG3′ and 5′CAATGCAAGAGGGAGTGTGA3′ for *Helena*, and 5′TTGACACTCAACTTCCGAACC3′ and 5′TTTCAAACCCCTGAATCTCG3′ for *Galileo*.

### Statistical Methods and Sequence Analyses

Most statistical analyses were performed using the statistical software SPSS version 19.0. The Kruskal and Wallis test [37] was done to see whether the differences in stability and transposition between hybrid families were significant. The Mann-Whitney test was used to compare genomic instability induced by transposition between hybrids and parental species.

For identification of AFLP marker sequences, a similarity was searched between the query sequences and TEs sequences of Genbank and Repbase [38] databases using the parameters defined by the CENSOR search tool (http://www.girinst.org/censor). The sequence data have been deposited in GenBank under the accession nos: JX997188; JX997190; JX997191; JX997192; JX997193; JX997194; JX997195: JX997196; JX997197; JX997198; JX997199; JX997200; JX997201; JX997202; JX997203; JX997204; JX997205; JX997206; JX997207; JX997208; JX997209; JX997210; JX997211; JX997212; JX997213; JX997214; JX997216; JX997217.

## Results

AFLP band patterns of 40 primer combinations were analyzed in each backcross generation and 28 in segmental hybrids. AFLP instability markers were selected in 3 backcross generations of hybrids (BC1, BC2, BC3) and in segmental hybrids between *D. buzzatii* and *D. koepferae*. Instability markers homologous to TE sequences by analysis in the data bases, were considered transposition markers, resulting probably from new insertions of TEs in the hybrid genome.

### Instability Markers

The number of different AFLP instability markers segregating in the genome of hybrids of BC1, BC2, BC3 generations was 30, 17, 25, respectively, and 11 in segmental hybrids. Its distribution by family was 19, in family 10; 26 in family 13, and 17, in family 40; the 10 remaining markers corresponding to other families where it was impossible to complete the 3 backcross generations (details Table S2). Parental species *D. buzzatii* and *D. koepferae* only showed 6 and 4 AFLP instability markers, respectively. The total number of instability markers segregating in the hybrid genomes was 83 (72 in interspecific hybrids and 11 in segmental hybrids) versus 10 markers in parental genomes. Among all instability markers, some corresponded to non repetitive sequences with homology to different regions of the *D. mojavensis* genome, and are supplied as supplementary material (Text S1). Comparisons of the total number of instability markers in BC3 show significant differences between hybrid families using a Kruskal & Wallis test (P = 0.01) (Table 1) but there were no significant differences between generations (not shown).

**Table 1:** Comparison of the number of instability markers in backcross 3 (BC3) between hybrid families.

	Kruskal-Wallis Test		
	χ^2^	k	P value
Total instability	8.761	2	0.010**
Instability by transposition	0.97	2	0.653

Sample size: 12, 12 and 16 flies for families 10, 13 and 40 respectively, χ^2^: chi square, k: degrees of freedom, **P≤0.01.

### Transposition Markers

**Figure 2:**
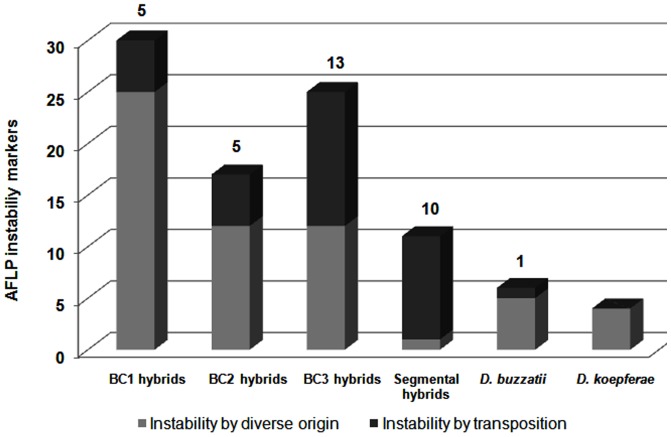
Markers in hybrids and parental species.

The number of transposition markers was 5, 5 and 13 in the BC1, BC2 and BC3 generations, respectively, and 10 in segmental hybrids (Figure 2). A total of 33 transposition markers were detected in the hybrid genomes whereas only one was found in the parental species (*D. buzzatii*). All except 3 transposition markers (CGGGG22, CACAT21 and TGCGG21) were longer that 200 bp (Table 2) and showed a wide range of similarity (0.1 to 0.94) with the TE sequences. In a few cases, transposition markers showed homology to internal or coding regions of one, or two TEs. This internal homology probably occurs because these TE regions are the most conserved in the database. The percentage of genomic instability due to transposition (number of transposition markers divided by the number of total markers in each backcross generation, multiplied by 100) was 16.6, 29.4 and 52.0% in the BC1, BC2 and BC3 generations, respectively.

**Table 2:** Sequences of AFLP markers showing homology to TE sequences.

*TEs*	*AFLP marker*	*Marker size*	*Pos*	*Sim*	*Homologous region*	*Hybrid*	*Genbank reference*
*Baggins1^#^*	*TGTCG21*	*377 bp*	*0.52*	*0.38*	*reverse transcriptase/RNAasa*	*SH*	JX997206
BATUMI*	*CACTC22*	*500 bp*	*0.60*	*0.60*	*polyprotein*	*SH*	JX997209
Bel_1*	*GGATT21*	*450 bp*	*0.93*	*0.93*	*internal region*	*BC1*	JX997190
*BS^#^*	*TGTCG22*	*443 bp*	*0.71*	*0.62*	*internal region*	*SH*	JX997207
CIRCE*	*CACTC21*	*529 bp*	*0.62*	*0.55*	*internal region*	*SH*	JX997212
DIVER*	*TGTCG23*	*415 bp*	*0.63*	*0.33*	*endonuclease/integrase*	*SH*	JX997208
Frogger*	*CACAT21*	*153 bp*	*0.61*	*0.41*	*internal region*	*SH*	not annotated
*Galileo^▪^*	*TGATT22*	*408 bp*	*0.99*	*0.1*	*internal region*	*BC3*	JX997200
*Galileo^▪^*	*CGGGG22*	*172 bp*	*0.75*	*0.60*	*internal region*	*BC3*	*not annotated*
Gypsy1*	*CAGCA21*	*491 bp*	*0.83*	*0.70*	*internal region*	*BC1*	JX997198
Gypsy1*	*CCCCC22*	*540 bp*	*0.83*	*0.83*	*internal region*	*BC1*	JX997192
Gypsy1*	*CCGAT22*	*264 bp*	*0.72*	*0.64*	*internal region*	*BC3*	JX997217
Gypsy1*	*TGATT21*	*554 bp*	*0.52*	*0.34*	*LTR*	*BC3*	JX997196
Gypsy3*	*CGAGT21*	*707 bp*	*0.45*	*0.27*	*polyprotein*	*SH*	JX997211
Gypsy8*	*TGTCG21*	*377 bp*	*0.45*	*0.32*	*internal region*	*SH*	JX997206
Gypsy8*	*TGATT26*	*505 bp*	*0.70*	*0.70*	*poliprotein*	*BC3*	JX997199
Gypsy9*	*CCGAT23*	*303 bp*	*0.48*	*0.38*	*internal region*	*BC3*	JX997204
Gypsy10*	*TGTCG23*	*415 bp*	*0.71*	*0.64*	*internal region*	*SH*	JX997208
Gypsy12*	*CGGCA21*	*1231 bp*	*0.60*	*0.47*	*internal region*	*SH*	JX997214
*Helena^#^*	*TGTCG22*	*443 bp*	*0.76*	*0.62*	*ORF2/endonuclease*	*SH*	JX997207
*Helena^#^*	*TGTCG27*	*441 bp*	*0.80*	*0.76*	*reverse transcriptase/RNAasa*	*BC2*	JX997216
*Helena^#^*	*TGTCG41*	*441pb*	*0.78*	*0.67*	*ORF2/endonuclease*	*DB*	not annotated
*Helitron-2^▪^*	*TGCGG21*	*145 bp*	*0.77*	*0.46*	*internal region*	*BC3*	not annotated
*Helitron-2^▪^*	*CGGCA21*	*1231 bp*	*0.62*	*0.51*	*5′region*	*SH*	JX997214
*Helitron-1N1^▪^*	*CCGAT22*	*264 bp*	*0.92*	*0.92*	*internal region*	*BC3*	JX997217
*HETA^#^*	*CACTC21*	*592 bp*	*0.41*	*0.31*	*internal region*	*SH*	JX997212
*Homo6^▪^*	*TGATT25*	*442 bp*	*0.62*	*0.50*	*internal region*	*BC2*	JX997193
*Homo6^▪^*	*TCAGT21*	*347 bp*	*0.88*	*0.88*	*internal region*	*BC2*	JX997194
*Homo6^▪^*	*TGATT27*	*441 bp*	*0.68*	*0.58*	*internal region*	*BC3*	JX997201
*Homo6^▪^*	*CCGAT24*	*320 bp*	*0.94*	*0.94*	*internal region*	*BC3*	JX997205
*LSU-rRNA_Hsa^Ø^*	*CAGCG23*	*448 bp*	*0.42*	*0.31*	*HSU 13369 locus*	*BC2*	not annotated
MAX*	*CACTC22*	*500 bp*	*0.66*	*0.50*	*polyprotein*	*SH*	JX997209
*MINI-ME^#^*	*GGCTC21*	*647 bp*	*0.58*	*0.46*	*internal region*	*BC1*	JX997188
*MINI-ME^#^*	*CCCCC21*	*540 bp*	*0.71*	*0.71*	*internal region*	*BC1*	JX997191
*MINI-ME^#^*	*TCAGT22*	*306 bp*	*0.71*	*0.62*	*internal region*	*BC2*	JX997195
Osvaldo*	*TGATT24*	*530 bp*	*0.63*	*0.51*	*internal region*	*BC3*	JX997197
Osvaldo*	*TGATT26*	*519 bp*	*0.56*	*0.46*	*internal region*	*SH*	JX997213
*Penelope^#^*	*CCGAT21*	*309 bp*	*0.91*	*0.13*	*internal region*	*BC3*	JX997203
*TART^#^*	*TGTCG23*	*415 bp*	*0.55*	*0.41*	*polyprotein*	*SH*	JX997208
*TART^#^*	*TCTCG21*	*218 bp*	*0.55*	*0.29*	*polyprotein*	*BC3*	JX997202
*Transib1^▪^*	*CAGCA22*	*359 bp*	*0.73*	*0.60*	*transposase*	*SH*	JX997210
*TRIM^#^*	*TGTCG23*	*415 bp*	*0.58*	*0.41*	*internal region*	*SH*	JX997208
*UHU^▪^*	*TGATT25*	*513 bp*	*0.66*	*0.52*	*internal region*	*BC3*	JX997198
ZAM*	*TGATT26*	*519 bp*	*0.58*	*0.35*	*internal region*	*SH*	JX997213

Sim: similarity between 2 aligned fragments using the parameters of CENSOR tool referenced in material and methods section, * LTR retrotransposon, ^#^ non-LTR retrotransposon, ^▪^ DNA transposon, ^Ø^ pseudogen, SH: segmental hybrid, BC1, BC2 and BC3:hybrids from backcrosses 1,2 and 3 respectively; DB: parental species *D. buzzatii.*

**Figure 3:**
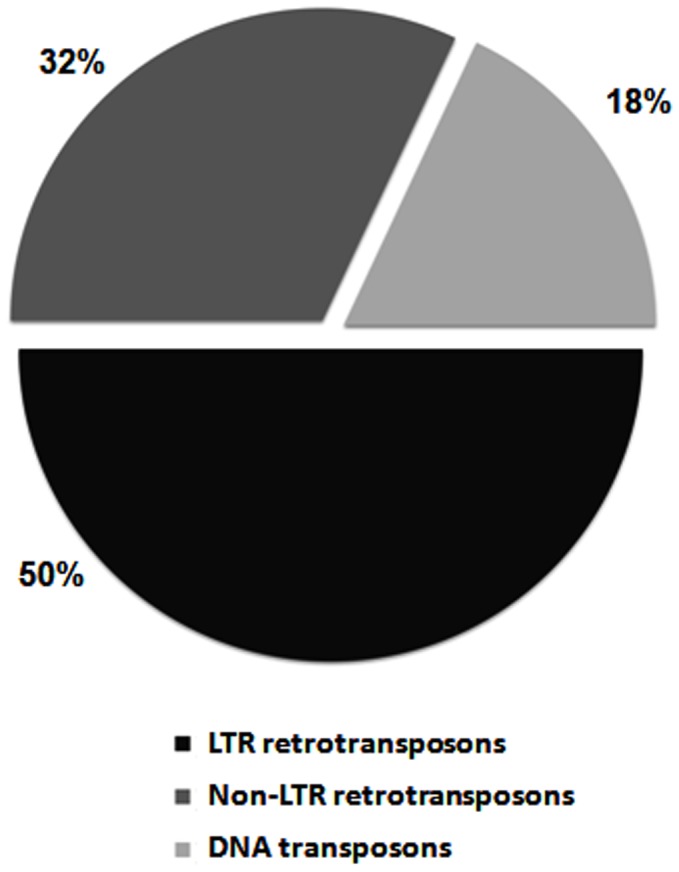
Percentage of transposition markers in hybrid.

Detailed analysis of the sequenced transposition markers allowed us to detect a pseudogene and 28 different TEs belonging to 14 TE families, including class I and class II elements (Figure 3). These TEs are probably responsible for most of the genomic instability in hybrids (Table 2). Three, 3 and 11 different TEs were detected in BC1, BC2, and BC3 generations, respectively, whereas 19 were identified in segmental hybrids. Interestingly, some specific TEs were detected in more than one generation. The genome of the parental species seems quite stable: only one element (*Helena*) was mobilized in *D. buzzatii* and none in *D. koepferae*.

No significant differences were detected between hybrid families for the number of markers associated to TEs (P = 0.65) in BC3 (Table 1). However, significant differences (P = 0.045*) were detected when the number of transposition markers between BC3 hybrids and parental species were compared (Table 3), bolstering the idea that the genome of hybrids induces a significant increment of instability by transposition. The value is only significant in family 40 (P = 0.035*) when analyzing each family separately, despite new TE markers have been detected in all of them.

**Table 3:** Comparison of the number of transposition markers between BC3 hybrid families and parental species.

Comparisons	N		Mann-Whitney Test	
	Hybrids	Parentals	U	P value
Hybrids vs.Parental species	40	30	513.5	0.045*
Family 10 vs.Parental species	12	30	170.5	0.286
Family 13 vs.Parental species	12	30	155.5	0.134
Family 40 vs.Parental species	16	30	187.5	0.035*

N: sample size, U: statistic value, *: P≤0.05; BC3: backcross 3.

### Transposition Rates

From the set of TEs detected by AFLP, *Osvaldo*, *Helena* and *Galileo* were studied by *in situ* hybridization (Table 4) and transposon display in parental species, but only by transposon display in hybrids. Although both techniques allow us to assess the number of euchromatic copies in the genome, transposon display has the advantage of amplifying both euchromatic and heterochromatic copies, whereas *in situ* hybridization only allows one to discern euchromatic copies, in spite of having been traditionally used to estimate total transposition rates in Drosophila [30], [39]. Both techniques were used for parental species analyses to estimate how many of the insertions detected by transposon display corresponded to heterochromatic insertions. Using FISH *Osvaldo* gave 5 euchromatic copies and a heavy stained centromeric signal in *D. buzzatii,* and only a centromeric signal in *D. koepferae,* whereas up to 40 and 39 copies were detected by transposon display in the 2 species, respectively. In the case of *Helena*, 5 and 12 euchromatic copies were detected by FISH in *D. buzzatii* and *D. kopeferae* respectively, but up to 28 and 25 copies by transposon display. *Galileo* had just one euchromatic copy in each parental species, and up to 43 and 45 copies by transposon display in *D. buzzatii* and *D. koepferae*, respectively.

**Table 4:** Chromosomal insertion sites of *Osvaldo*, *Helena* and *Galileo* in parental species.

Element/Species	Chromosomes					
	X	2	3	4	5	Total
***Osvaldo***						
*D. buzzatii*	G2, C	E4, D4	D1, C	C	C	4
*D. koepferae*	C	C	C	C	C	0
***Helena***						
*D. buzzatii*	A2	E2	G1, C		A5, D2	5
*D. koepferae*	A2, F3, G2	B2, E2, F1		D4, G2	C4.1,C4.2,C5, F2	12
***Galileo***						
*D. buzzatii*				E3		1
*D. koepferae*					G1	1

C: centromere.

In view of its advantage the transposon display technique was used to estimate the transposition rates in hybrids and parentals species for each element in each family and backcross generation (Tables S1).

### *Osvaldo* Transposition Rates

The bands of *Osvaldo* detected by transposon display ranged in size from 171 to 1177 bp, each insertion including 152 bp of a 5′ LTR fragment (determined after the restriction map of this TE) and its flanking genomic region (variable in size depending on the genomic insertion site of each copy). The number of insertions in hybrid genomes ranged between 35 (BC1 family 40) and 54 (BC3 family 10), showing new insertions in all families (e.g. 48 in BC3 of families 1 and 13) except family 40 (BC1). However, only one new insertion was observed in the family B8 in the parental species *D. buzzatii* (Table 5). The basal transposition rates of *Osvaldo* estimated for *D. buzzatii* and *D. koepferae* ranged from 0 to 3.3×10^−3^. The transposition rate of *Osvaldo* increased by one order of magnitude (10^−2^) in hybrid families compared to parental species; this was observed in the 3 backcross generations, although not in all hybrid families (Table 6). The number of new insertions in the 3 hybrid backcrosses was significantly higher in hybrids than the parental species (Table 7), with differences between hybrid families in BC1 and BC3 (Table 8).

**Table 5:** Number of *Osvaldo*, *Helena* and *Galileo* insertions detected by transposon display.

HYBRIDS	BC1			BC2			BC3		
	Totalinsertions	Newinsertions	Ni	Totalinsertions	Newinsertions	Ni	Totalinsertions	Newinsertions	Ni
***Osvaldo***									
Family 1	40	3	9	40	3	7	48	7	53
Family 10	38	1	1	48	9	30	54	11	60
Family 13	38	1	1	38	1	2	48	11	22
Family 40	34	0	0	40	5	10	50	11	46
***Helena***									
Family 1	18	0	0	23	3	11	22	2	14
Family 10	18	0	2	22	2	5	25	1	4
Family 13	17	2	2	22	0	0	24	1	6
Family 40	21	0	0	24	1	2	25	1	3
***Galileo***									
Family 1	25	1	5	30	0	0	32	2	2
Family 10	35	0	0	39	0	0	40	0	0
Family 13	48	0	0	47	0	0	49	1	4
Family 40	52	2	7	55	1	5	56	0	0
**PARENTALS**	***Osvaldo***			***Helena***			***Galileo***		
	**Total** **insertions**	**New** **insertions**	**Ni**	**Total** **insertions**	**New** **insertions**	**Ni**	**Total** **insertions**	**New** **insertions**	**Ni**
***D. buzzatii***									
Family B4	40	0	0	27	1	6	41	0	0
Family B8	35	1	3	28	0	0	43	0	0
***D. koepferae***									
Family K3	39	0	0	25	0	0	42	0	0
Family K9	34	0	0	22	0	0	45	0	0

Ni: insertions copy number; BC1, BC2 and BC3 correspond to backcrosses 1, 2, 3 respectively.

**Table 6:** Transposition rates of *Osvaldo, Helena* and *Galileo* in hybrid families and parental species.

HYBRIDS	BC1							BC2								BC3								
	Ni		N	A		TR		Ni		N		A		TR		Ni			N		A		TR	
***Osvaldo***																								
Family 1	9		7	37		1.7×10^−2^		7		18		37		5.2×10^−3^		53			40		41		1.6×10^−2^	
Family 10	1		2	37		6.7×10^−3^		30		18		39		2.1×10^−2^		60			25		43		2.7×10^−2^	
Family 13	1		2	37		6.7×10^−3^		2		15		37		1.8×10^−3^		22			36		37		8.3×10^−3^	
Family 40	0		5	34		0		10		14		35		1.0×10^−2^		46			18		39		3.2×10^−2^	
***Helena***																								
Family 1	0		7	18		0		11		17		20		1.6×10^−2^		14			40		20		8.8×10^−3^	
Family 10	0		2	18		0		5		19		20		6.5×10^−3^		4			33		24		2.5×10^−3^	
Family 13	2		3	17		1.9×10^−2^		0		16		22		0		6			38		23		3.4×10^−3^	
Family 40	0		5	21		0		2		13		23		3.3×10^−3^		3			16		24		3.9×10^−3^	
***Galileo***																								
Family 1	5		7	24		1.4×10^−2^		0		18		30		0		2			40		31		8.0×10^−4^	
Family 10	0		2	35		0		0		17		39		0		0			30		40		0	
Family 13	0		3	48		0		0		11		47		0		4			32		48		1.3×10^−3^	
Family 40	7		5	52		1.4×10^−2^		5		14		55		3.3×10^−3^		0			24		56		0	
**PARENTAL SPECIES**																								
	***Osvaldo***								***Helena***									***Galileo***						
	**Ni**	**N**			**A**		**TR**		**Ni**		**N**		**A**		**TR**		**Ni**			**N**		**A**		**TR**
***D. buzzatii***	3	28			74		7.2×10^−4^		6		28		54		2.0×10^−3^		0			26		84		0
***D. koepferae***	0	24			73		0		0		19		47				0			24		87		0

Ni: new insertions copy number, N: number of individuals, A: number of original insertions, TR: Transposition rate; BC1, BC2 and BC3 correspond to backcrosses 1, 2, 3 respectively.

**Table 7:** Comparison of the number of new insertions between hybrids and parental species by Mann-Whitney test.

	Parentals	BC1			BC2			BC3		
TEs	N	N	U	P value	N	U	P value	N	U	P value
***Osvaldo***	53	16	206.5	<10^−3^ **	65	1170.5	<10^−3^ **	119	1330.5	<10^−3^ **
***Helena***	47	17	372	0.439	65	1440	0.420	127	2772	0.278
***Galileo***	53	17	185	<10^−3^ **	60	1431	0.031*	126	3180	0.107

TEs: transposable elements, N: number of individuals, U: statistic value, *:P≤0.05.

**: P≤0.01; BC1, BC2 and BC3 correspond to backcrosses 1, 2, 3 respectively.

**Table 8:** Comparison of the number of new insertions between hybrid families by Kruskal-Wallis test.

		BC1		BC2		BC3	
TEs	N	χ^2^	P value	χ^2^	P value	χ^2^	P value
***Osvaldo***	4	31.18	<10^−3^**	6.206	0.102	10.877	0.012*
***Helena***	4	4.667	0.198	8.911	0.031*	1.108	0.775
***Galileo***	4	10.768	0.013*	17.623	0.001**	6.875	0.076

TEs: Transposable elements; N: number of families, U: statistic value,

*:P≤0.05; P≤0.01; BC1, BC2 and BC3 correspond to backcrosses 1, 2, 3 respectively.

When transposition rates were calculated in hybrid males and females separately, a trend of increasing rates was seen in males, from families 13 and 1, compared to females (Table S3), but differences were only statistically significant for family 1 (P = 0.01*). Because hybrid females had been repeatedly backcrossed with *D. buzzatii* males, transpositions always occurred in the hybrid female germline, suggesting (but not proving) that the increase of transposition rates in males could be due to a higher male somatic transposition rate in these 2 families.

### *Helena* Transposition Rates

In the case of the *Helena* element, we analyzed the 3′ end, obtaining insertion sizes ranging from 110 to 630 bp. These fragments included 107 bp of the 3′ *Helena* end plus a flanking DNA sequence. The number of insertions observed per hybrid family ranged between 17 and 25 (Table 5), some of which were new; e.g. 3 new insertions were found in BC2 from family 1, but only one insertion in the family B4 of *D. buzzatii.* The basal transposition rates of *Helena*, ranged from 0 to 8.2×10^−3^ for *D. buzzatii*, and 0 for *D. koepferae*. *Helena* showed an increase of transposition (10^−2^) in some families of the BC1 and BC2 generations, but this activity decreased to the basal transposition rate in the BC3 generation (Table 6). However, the number of new insertions in hybrids compared to parental species did not reach significance at statistical level (Table 7), neither were the differences between families, except in BC2 (Table 8). Comparisons of *Helena* transposition rates between males and females do not show a clear trend because the transposition increase was seen in males of a family and females of another.

### *Galileo* Transposition Rates

The amplified insertions of *Galileo* contain a fragment of 182 bp of the 5′ end and a flanking DNA sequence ranging from 191 to 866 bp. Up to 7 new insertions (family 40, BC1) were found in the genome of hybrids, but no new insertions were detected in parental species (Table 6). While the basal transposition rate of *Galileo* for parental species is 0, this element shows high transposition activity (≈10^−2^) in 2 families of the BC1 generation showing significant differences from the parental species (Table 8), decreasing later in BC2 and BC3 (Table 6). When families were compared (Table 8) significantly differences in transposition rates were seen in BC1 and BC2, showing that the effect of hybridization on TE instability depends on the families analyzed.

Statistical analyses showed significant differences (P<10^−3^) of *Osvaldo* transposition rates between hybrids and parental species in the 3 backcrosses. In the case of *Helena* and in spite of the new insertions being detected in hybrids compared with parental species (only one new insertion in *D. buzzatii*, Tables S1), the differences were not significant (Table 7). For the *Galileo* element, significant differences were found in BC1 and BC2 (Table 7).

### Expression of *Osvaldo*, *Helena* and *Galileo*

Transcription of *Osvaldo, Helena* and *Galileo* was examined in the testes, ovaries and carcasses of parental species *D. buzzatii* and *D. koepferae* by RT-PCR. *Osvaldo* and *Helena* were expressed in germinal and somatic tissues isolated from adult males and females. In the case of *Galileo*, no transcriptional activity was detected in germinal or somatic tissues of both species *D. buzzatii* and *D. koepferae* (Figure S1) suggesting that this element cannot move autonomously in the genome of our stocks, but probably needs assistance from a second TE for its mobilization.

## Discussion

The joint analysis of AFLP patterns in 3 hybrid families, a strain of segmental hybrids and 4 families of parental species, shows the existence of genomic instability in the hybrid genomes, revealed by 72 instability markers plus a pseudogene. In contrast, only 4 and 6 instability markers were found in *D. koepferae* and *D. buzzatii* parental genomes, respectively. A high percentage (16, 29, 52 and 90% in BC1, BC2, BC3 and segmental hybrids, respectively) of them showed homology with TEs in hybrids probably originated by the TEs whose mobilization produce new insertions detected as new bands in the AFLP pattern. Another fraction of instability markers had homology with the *D. mojavensis* genome (the Drosophila sequenced genome phylogenetically closer to the species considered here) and probably originated by the suppression of a restriction site and/or by double-strand breaks that are usually associated to the loss of chromosomal segments or the rearrangement of genetic material [40].

It is noteworthy that 90% of the markers detected in segmental hybrids had homology with TE sequences. Because these hybrids have a portion of chromosome 4 (as detected by chromosomal asynapsis) of *D. koepferae* introgressed in the *D. buzzatii* genome, this region could be involved in TEs instability rendering this line unstable. This line was also maintained for 60 generations which would explain the accumulation of new TE copies. Hybrid families from BC3 generation and parental species showed significant differences in transposition instability (P = 0.045), but when families were considered separately, differences were only significant for family 40, despite all families showed an increase of transposition markers compared to parental species (only one new transposition marker detected). This result is probably due to the highest number of hybrids analyzed in this family and/or to an increasing segregation of instability markers only in certain hybrid families. Indeed, in previous studies *Osvaldo* transposition bursts have been preferentially found in some interspecific hybrids of *D. buzzatii* and *D. koepferae* [41]. The overall increase of genomic instability induced by transposition, as found in Drosophila interspecific hybrids, suggests that the stress produced by hybridization induces a significant activation of transposition in hybrid genomes. These observations are in agreement with studies on genomic instability of natural hybrids of *Amaranthus*, where the new AFLP hybrid markers detected in hybrids have homology with TEs associated to the mobilization of repetitive DNA [42]. Moreover, in the present study an increase of genomic instability in early generations of backcrossing was also observed. This is not uncommon because the level of introgression in the first backcross generation is the highest, decreasing in the next generations due to the increment of the proportion of the *D. buzzatii* genome through the repeating backcrossing with a *D. buzzatii* parental male. These results are in accord with those of Madlung *et al.* [43] who detected hybrid instability at the early generations of *Arabidopsis* interspecific crosses.

*Osvaldo* [41] is an active retrotransposon expressed in somatic and germinal tissues of *D. buzzatii* and *D. koepferae* stocks that we used. I*n situ* hybridization, transposition rates and expression of *Helena* element, bolster the notion that this element is also an active element in the genomes of *D. buzzatii* and *D. koepferae* that has the capacity to move and create new insertions. An element that deserves special mention is *Galileo,* where no expression of transposase was found, suggesting that, in our stocks, most copies could be deleted showing passive activity mediated by a cooperative TE. New copies of *Galileo* have been noted in the hybrid genomes whose nucleotide sequences, amplified by transposon display, often contain other TE fragments in the same sequences, suggesting that their transposition may correspond to the activity of other autonomous TEs carrying inserted fragments of *Galileo*. Interestingly, something similar occurs when this element is screened in the genome of *D. mojavensis*, in which fragments of *Galileo* are frequently located next to other TEs.

Several attempts to induce transposition in Drosophila through hybridization have been attempted without success [26], [44] Nowadays, numerous examples of hybrid instability associated to TE activity are found in plants [23], [45] and, to a lesser extent, in insects [46], [47] and mammals [28], [29]. Our study constitutes the first genome-wide survey of hybrid instability showing that a high proportion of the instability markers detected in hybrids correspond to TE. Thus, an average of 32 and 90% of the markers detected in the three backcross generations and the segmental hybrids, respectively, correspond to a wide variety of TEs. The estimated transposition rates for *Osvaldo* and *Helena* in hybrids are higher than spontaneous estimated for some TEs in natural populations of Drosophila (10^−4^) [48], which can be directly associated to the genomic instability in the hybrid genomes. TEs have the capacity to create new copies in the genomes through many mechanisms, including excision, replication, insertion, and ectopic recombination. In *D. melanogaster*, for example, new chromosomal rearrangements and mutations can be produced by ectopic recombination between different copies of the *hobo* element [49], and in humans, ectopic recombination between *Alu* sequences seem to be important in producing deleterious mutations [50]. In our study, the new *Galileo* copies could also be attributed to ectopic recombination of *Galileo* long TIRs [51]; we consider that the new copies of the 3 TEs analyzed were probably produced by transposition because here a high number of copies observed were detected in heterochromatic regions, in which recombination rates are usually low. Elements outside heterochromatin are often active, hence more than 50% of mutations in Drosophila have been assigned to direct TEs insertion [52]. However, despite euchromatin harboring most of Drosophila genes, the effect of recombination cannot completely be ruled out because many genes are also found in heterochromatic regions [53].

In previous experiments, increases in transposition rate of *Osvaldo* were seen in interspecific hybrids (10^−2^) compared to parental species *D. buzzatii* (10^−3^) [54]. These results are in agreement with the transposition rates estimated for *Osvaldo* in the 3 backcross generations (10^−2^) and confirm its higher ability to mobilize in the hybrid genomes compared to its lower transposition rate in the parental species *D. buzzatii* (10^−3^) and *D. koepferae* (<10^−3^). However, in family 13 the transposition rate of *Osvaldo* is of the order of 10^−3^ in all backcross generations which supports the idea that bursts of transposition do not occur equally in all families of hybrids. The same was observed for *Helena* and *Galileo,* where differences in transposition rates were found between generations of backcrosses and hybrid families. This result is unsurprising considering that we introduce the genome of *D. buzzatii* in each backcross generation; despite the amount of the genome being introduced is the same, the region introgressed is different in each hybrid family. It is known that the mobility of some TEs is controlled by specific genomic loci; for example, *gypsy*, *Idefix* and *Zam* are controlled by the locus *flamenco* [55], [56] and *P* elements by a subtelomeric region of X chromosome [57]. These loci comprise fragmented and imperfect copies of retrotransposons that are the precursors of PIWI-interacting RNAs (piRNA) responsible of post-transcriptional TE control. Another result of note is the different activity of the 3 elements analyzed in hybrids; the most active element being *Osvaldo,* followed by *Helena* and then *Galileo*. These differences were also found across generations, indicating that a variation in TE stability exists depending on the element and the genetic background of the hybrid genome. In *D. melanogaster*, for example, there is a natural variation in TE stability depending on the genetic background [58]. In our case we hypothesized that TEs activation in hybrids could occur in a similar way to that in dysgenic crosses, due to the lack of specific maternal piRNAs Another hypothesis, not exclusive, could be the divergence of genes involved in the piRNA pathway. Recent findings in hybrids between *D. melanogaster* and *D. simulans* attributed the derepression of different TEs to divergence in piRNA genes rather than species-specific differences in piRNAs derived from TEs [47].

It is of note that, in the case of the *Osvaldo* retrotransposon, a trend towards an increase in transposition rate in hybrid males occurs. In other retrotransposons, e.g. *copia* element, transpositions are limited to male spermatocytes [59]. However, since hybrid males do not participate in hybrid crosses, we suggest that differences of transposition between sexes could be due to an increase of transpositions in the somatic line of males. Malone et al. [60] suggested a tissue-specific regulation of certain element classes correlated with tissue specific expression of piRNA clusters (genomic loci producing significant levels of piRNA). In *Xenopus,* a dramatic increase in the number of misexpressed genes found in hybrid females, compared to testes of hybrid males, suggests that divergence in female expression may be involved in sterility of hybrid males due to the inherent sensitivity of spermatogenesis [61]. We hypothesize that misregulation of genes implicated in the piwi pathway contribute to the instability of TEs in germinal lines. However, it is difficult to provide a valid explanation because we ignore the regulation mechanisms of *Osvaldo* and other elements.

Because different classes of TEs have been mobilized in hybrids, they could contribute to the genome reorganization either by transposition or/and ectopic recombination, producing deletions, duplications and inversions. Previous studies in *D.buzzatii-kopeferae* hybrids showed a high frequency of new chromosome rearrangements induced by introgressive hybridization [62]. Overall, our study contributed to a better understanding of the effects of hybridization in transposition release in hybrids, but the mechanisms triggering transposition are poorly understood and more knowledge about them would be of great importance to demonstrate that transposable elements are involved in speciation. Knowledge concerning the expression rates and TEs transcripts location could be the next step towards understanding the molecular mechanisms activating TEs in Drosophila hybrids.

## Supporting Information

Figure S1 **RT PCR of Osvaldo, Helena and Galileo in somatic and germinal tissues of** ***D. buzzatii*** **and** ***D. koepferae*** **.**
(TIFF)

Tables S1 **Osvaldo, Helena and Galileo matrices of transposon display experiment.** Asterisks represent new insertions. The presence of an insertion is represented by 1 and the absence by 0.
(DOC)

Table S2 **Number of AFLP markers observed in each family and backcross generation.**
(DOC)

Table S3 **Transposition rates for male and females BC3 hybrids.**
(DOC)

Text S1 **AFLP instability markers with no homology to transposable elements.**
(DOC)
