# Supplementary figures and images for: A Genome-Wide Survey of Genetic Instability by Transposition in Drosophila Hybrids
Source: PLoS One. 2014 Feb 20;9(2):e88992. doi: 10.1371/journal.pone.0088992 (PMC3930673; doi:10.1371/journal.pone.0088992)

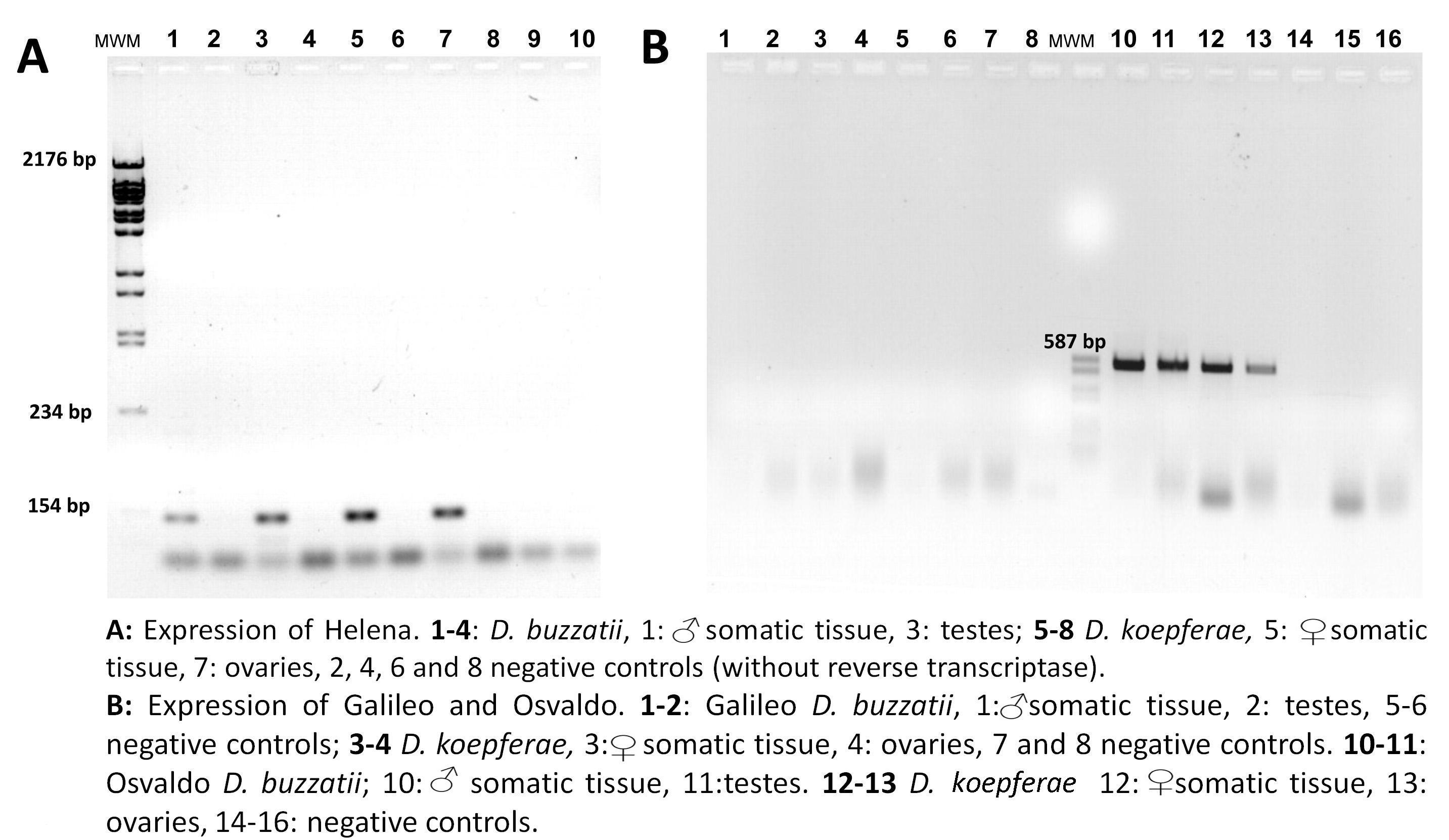

Supplement: Figure S1 — RT PCR of Osvaldo, Helena and Galileo in somatic and germinal tissues of D. buzzatii and D. koepferae . (TIFF) [file pone.0088992.s001.tif]
