# Supplementary material for: A Genome-Wide Survey of Genetic Instability by Transposition in Drosophila Hybrids
Source: PLoS One. 2014 Feb 20;9(2):e88992. doi: 10.1371/journal.pone.0088992 (PMC3930673; doi:10.1371/journal.pone.0088992)
Supplement: Table S2 — Number of AFLP markers observed in each family and backcross generation. (DOC) [file pone.0088992.s003.doc]

| **Table S2: Number of AFLP markers observed in each family and backcross generation** | | | | |  | |
| --- | --- | --- | --- | --- | --- | --- |
| **AFLP instability markers**  Family identification | | Backcrosses |  |  | |  |
|  | BC1 | BC2 | BC3 |  | |  |
| 10 | 7 | 4 | 8 |  | |  |
| 13 | 7 | 12 | 7 |  | |  |
| 40 | 6 | 1 | 10 |  | |  |
| 7 | 3 |  |  |  | |  |
| 12 | 1 |  |  |  | |  |
| 23 | 2 |  |  |  | |  |
| 34 | 1 |  |  |  | |  |
| 39 | 3 |  |  |  | |  |
| **AFLP transposition markers**  Family identification Backcrosses | | |  |  | |  |
|  | BC1 | BC2 | BC3 |  | |  |
| 10 | 0 | 0 | 4 |  | |  |
| 13 | 2 | 4 | 5 |  | |  |
| 40 | 2 | 1 | 4 |  | |  |
| 34 | 1 |  |  |  | |  |
| Markers shared between backcrosses (BC) and / or families are counted only once | | | | | | |
