# Supplementary material for: A Genome-Wide Survey of Genetic Instability by Transposition in Drosophila Hybrids
Source: PLoS One. 2014 Feb 20;9(2):e88992. doi: 10.1371/journal.pone.0088992 (PMC3930673; doi:10.1371/journal.pone.0088992)
Supplement: Table S3 — Transposition rates for male and females BC3 hybrids. (DOC) [file pone.0088992.s004.doc]

| **Table S3: Transposition rates for male and females BC3 hybrids** | | | | | |
| --- | --- | --- | --- | --- | --- |
|  | Ni | 2 | N | A | TR |
| **Osvaldo** |  |  |  |  |  |
| Family 1 | 53 | 2 | 40 | 41 | 1.6x10^-2^ |
| ♀ | 15 | 2 | 20 | 41 | 9.1x10^-3^ |
| ♂ | 38 | 2 | 20 | 41 | 2.3x10^-2^ |
| Family 10 | 60 | 2 | 25 | 43 | 2.7x10^-2^ |
| ♀ | 25 | 2 | 11 | 43 | 2.6x10^-2^ |
| ♂ | 35 | 2 | 14 | 43 | 2.9x10^-2^ |
| Family 13 | 22 | 2 | 36 | 37 | 8.2x10^-3^ |
| ♀ | 3 | 2 | 19 | 37 | 2.1x10^-3^ |
| ♂ | 19 | 2 | 17 | 37 | 1.5x10^-2^ |
| Family 40 | 46 | 2 | 18 | 39 | 3.2x10^-2^ |
| ♀ | 19 | 2 | 7 | 39 | 3.4x10^-2^ |
| ♂ | 27 | 2 | 11 | 39 | 3.1x10^-2^ |
| **Helena** |  |  |  |  |  |
| Family 1 | 14 | 2 | 40 | 20 | 8.7x10^-3^ |
| ♀ | 10 | 2 | 20 | 20 | 1.2x10^-2^ |
| ♂ | 4 | 2 | 20 | 20 | 5.0x10^-3^ |
| Family 10 | 4 | 2 | 33 | 24 | 2.5x10^-3^ |
| ♀ | 1 | 2 | 19 | 24 | 1.1x10^-3^ |
| ♂ | 3 | 2 | 14 | 24 | 4.4x10^-3^ |
| Family 13 | 6 | 2 | 38 | 23 | 3.4x10^-3^ |
| ♀ | 4 | 2 | 19 | 23 | 4.5x10^-3^ |
| ♂ | 2 | 2 | 19 | 23 | 2.2x10^-3^ |
| Family 40 | 3 | 2 | 16 | 24 | 3.9x10^-3^ |
| ♀ | 0 | 2 | 8 | 24 | <10^-3^ |
| ♂ | 3 | 2 | 8 | 24 | 7.8x10^-3^ |
| **Galileo** |  |  |  |  |  |
| Family 1 | 2 | 2 | 40 | 30 | 8.3x10^-4^ |
| ♀ | 2 | 2 | 20 | 30 | 1.6x10^-3^ |
| ♂ | 0 | 2 | 20 | 30 | <10^-3^ |
| Family 10 | 0 | 2 | 30 | 40 | <10^-3^ |
| Family 13 | 4 | 2 | 32 | 48 | 1.3x10^-3^ |
| ♀ | 0 | 2 | 18 | 48 | <10^-3^ |
| ♂ | 4 | 2 | 14 | 48 | 2.9x10^-3^ |
| Family 40 | 0 | 2 | 24 | 56 | <10^-3^ |
| Ni: new insertions, N: sample size, A: original insertions, | | | | | |
| TR: Transposition rate | | | | | |
