# Supplementary material for: A Genome-Wide Survey of Genetic Instability by Transposition in Drosophila Hybrids
Source: PLoS One. 2014 Feb 20;9(2):e88992. doi: 10.1371/journal.pone.0088992 (PMC3930673; doi:10.1371/journal.pone.0088992)
Supplement: Text S1 — AFLP instability markers with no homology to transposable elements. (DOC) [file pone.0088992.s005.doc]

**Text S1: AFLP instability markers with no homology to transposable elements**

**CGGCA21**

GCACATCGTCGTCAATGCCATTGAGCGCACAATAACCGATTGGTTGCAGCCGGTGGTGGATCGTAGCATACGCATTGCATGCGCCACAACTGAACAGATCATACGTAAGGATTTCGCATTGGACGCGGATGAGAATCGTATGCGCACTGCAGCCCATCAGATGGTGCGCAATCTAGCCGCTGGCATGGCTATGATAACCGGAAAGGACGAGATCGCACGGGCGATTAGCCAGAACTTGCATAAGGCATTCATGGCCGCGTTGACGGGTGTATCGAGCATGTCCGACATACAGGCAGCATCGATGCAGCTGGCAAACGAGAATGTGGAATTGGTTTGTGCATTTATACAGAAGACATCGGCTGAGAAGTCGGCGCTCGAGATCGATCGACGCCTGTCGACCGACTTTGAGACAAGGAAAATTGCACGCGAAGAGGGCAGCCGATTTGTTGATGCACAAATCTTGAGCTATCAGCAGGAGCGTCTGCCGGAGCCAGTGCGTCTGAAGGTGGGTGCAGCCCCACCCACACTGTACGCCGTCTATTCG

**CGGGG21**

CGTTGGCCAAAAACCAAAATGCCAGCCGTTAGCAGCATGTGCAGTCAACTGGCTGGCATTTCGGCAAATACATTCGTAATTCAAATGCAAATTTTTGGCAAAAGAAAAGTATTTTTTTTTCTGTTCTTTTCGTTCGTGTGGCCGTAAATTGTGAAACAGCTGCGC

**GTCTC21**

TATGGCGCCACCGAGGGCAATGCGAACATTATGAATCATGACAACACAGTGGGCGCCATTGGTTTTGTGTCGCGCATATTGCCCAAGGTTTATCCCATTTCAATAATACGCGCCGATCCCGATACGGGCGAGCCCATACGCAACAAGGATGGCCTCTGCGAACTGTGCGAGCCCAACGA

**GGCTC22**

GCCGTTGATAAAAACCTGTGGTATGGTTGGCCAGTCGGTGTAGTCTTTGATGCCTGTGCGAGAGTGGCAGAAATTTAGTTAGATTTCGGTAACTTGAGCTGTGATCTCACCCACCTTGACGCAGTGCCTCGTTCTGCAGAACATCATGTGCATCGTATTGTACACCATGCATGCGCATGATCTGCACCACAGCATTGCTGAAGCCGCAGCGCGGAGCCTGTGGATTGCCCTTCATGAATACGACCACTTTGTTGGTGCGCACCAGTTTGTCCAGTGTCTCCTTGTCCAATGCGGCCGGCGGAGCAGCTGCATCCGCGCTGTAGAAGCGTGGAGATGTTGCCAGCGCTGGCATCGTGCGGATTGTCGTTGCCAAATTGTTGGCTAGGCTCGGTCGCAGCAAGATTTGGCAAATTTTGTTCATTTTTGCTTATATATTACTGTGTATTTCCACGCACGACTTTTTATGAATTGGAAATAATTTTGTTTTATAATGTGTTGTGTGTTATGTTGACGCAATAAGCTCTAACATATGTGCATTGACGCAATTTTGCGAGCAAGGCTCCCTAGTGCTCCTCACTGTTACAAATTCTACTATGACACATGACGTTGGGCTCTGCTCTGTTAGTTTGAATTTGAAT

**CAGCG21**

CATGATCAGCGAGCGGAAGGTGTGGGCGTAGTTGAATTTCTGGCACTGTCGCATCGCATAGTTGGCCTCGTCATAGGCGACCAGCACATAGCACTGATCGCCCGAGACGCTCGTGTTCTCGGCCCCGCCATAGGCGCTCGTGTCGCACCAGTACTCTTCATTGGGATACAGCTCGGGGGCTGTGAATATGATGAGCGCCATGAACCAGGCAGCTGGCAGCTTGCACAGAAAGATTATCAGCACGGAGCGCAGCTGCCAGATGCCAAAGTCACCCAGGACTTGCGAGATGACATCTTCATCTGCAGTCTCTGACATCTCCTCCTCCTCCTGCTGATGCTCAGCATCCTGCTGGCTGCTGTATATATAGCTCAGGTGGGCGTGTGCCTCTGGCGTGGGCGTTGGTCGGCGCTTTTGCCACTTGGCCTCGGCGGGACTGTAGCTTTGATGCATTCTCCCATGCGTTCAATCGAGTAACCACGTCTTACGCGCTTCAAACCTCCAGCATAACTGCGACCACGC

**GCATT21**

GCCGTTGATAAAAACCTGTGGTATGGTTGGCCAGTCGGTGTAGTCTTTGATGCCTGTGCGAGAGTGGCAGAAATTTAGTTAGATTTCGGTAACTTGAGCTGTGATCTCACCCACCTTGACGCAGTGCCTCGTTCTGCAGAACATCATGTGCATCGTATTGTACACCATGCATGCGCATGATCTGCACCACAGCATTGCTGAAGCCGCAGCGCGGAGCCTGTGGATTGCCCTTCATGAATACGACCACTTTGTTGGTGCGCACCAGTTTGTCCAGTGTCTCCTTGTCCAATGCGGCCGGCGGAGCAGCTGCATCCGCGCTGTAGAAGCGTGGAGATGTTGCCAGCGCTGGCATCGTGCGGATTGTCGTTGCCAAATTGTTGGCTAGGCTCGGTCGCAGCAAGATTTGGCAAATTTTGTTCATTTTTGCTTATATATTACTGTGTATTTCCACGCACGACTTTTTATGAATTGGAAATAATTTTGTTTTATAATGTGTTGTGTGTTATGTTGACGCAATAAGCTCTAACATATGTGCATTGACGCAATTTTGCGAGCAAGGCTCCCTAGTGCTCCTCACTGTTACAAATTCTACTATGACACATGACGTTGGGCTCTGCTCTGTTAGTTTGAATTTGAAT

**CGCCC22**

GCTTTACCCTCTGGACCAATGTACGTCACTCCTCGCATCGCATCCTGCCAATACGGCTTTGAGATTCGATAGATGCTACATGGCTTTGAATGACACATCGCCAGCCTGTTGCCGGTGGCCGTCGCCGCGATGCACTTCAGACAGTCATCGCTAAGCAGCGGAGTGCCTAACGCAGCTGAAATCAGTGAACAATGAATATGAATGGGATGATGATATATATATATATATGTAATTATTCGTAATTGTTCAACAGTTTCGGCATCAAAT

**CGGAT21**

CGTTCAATGGGTTCAGGGATTTTTTATTTGCACTTGTCGCTTGCCACTGCTCGCCGGTCTGTCTATATGCCTGCCTTACCGTCCTTCCGCTTGGTCACTCTAAAAATAGCTCAGCGGCACTTGCGACTTCACATTTGTTGTCGCACTCACGCTCCCTTTATTTGCCATTCTGTTGCTCTGCGTCGCTTGCTGGGGTATCCGTAGGTGCCCGTGTGAGCAAATACATCTGATTTTGGATGCTAAATGATAAACTGCCTCACTGCAAGCAGAGGACACAGTTTGGGTCTGTTGCGTCGACATTGTGCTCAATTGACAACGAGCCTCATTTTGATGCCGTGTGTGTGTAACATTTCGCATGTCGTCCTTGACCCAAAGCTGCGCCGGTAAATAAAGAATCGACGCCTTGCCTAACAGTTTCCACTCACACACATATACATAAACACACACATACATACATACTAATGTACATATAAATGTATTTATTACCAGCCTCACCACCCCAGGCAACGTAACTGGG

**CGGCG21**

GCACCTTTGCATACACAAATAGGGGGCTGCGGCCCGGACAGCTAGGGAGGGGGGCGTGTGGTTATAAATTTACTCAGGACTCATCACTGACTGCGTACCAATTCGCATAGAAGCCGAATCTGTTTACATATCAATTGGCATTGCTTACCTAAAAGAGAAGAGACAGAAATGAAACATTAGAAATTTATTTGGAAAATTCTATATATATTTATATATATATATATATATACTTTGTATATATGTACCTAGATCATGCAGAAT

**GTCGG21**

GCCGGCCGCCGACTGCGGTGCGGTAAGTAATATTTACACATACATATATGCATATACATGCACACTGCTCTCACCCGCTTTGTGTTTGCTCAAATATTGCACTCTAACTCCTTCTACCGCAAATGGTTATCCTACTTTTGGCGTTGCCGTACTCCCATTTGGGACACCCTATGGACTGCCTGTGACTTTCAGCCACTCTGTGTTTATTCAAATATTGCACTCTGAAACCCTCTACCGCAAACGGTTATCCCACTTTTAGCGTTGCCGTATTCCCATTAGGGACTGCCCTATGACTTCATCAACCACCGCGGCGCAACGCAACAGACCCAAATGGTAAATATATGTATGTTGCACCACCCCCTCTTTTATGTCGCATTTCTTACGTTTGAAGATATTGTAAGTATGTGTGTGTAAGTGTTGGTGTGTATGAAAGGCACTCAATTTAGCAGGCAGAGCGCTGGAAAAAATCGCGAAACGCGACCTCGACGCAGTCATCGGCGTTGCCCATCTTCAATGACGCCCACTGTGATGTCATCGTGTCACTTGCTCTTTTATTGGGAGCGCTGTCATTGACTAGCCACACAAATACACACGAACATATACTCATACACACTCGCACAGCAAGAAGCAACACTATGTATCAATTTTCATTAGGGGTTTCGTTTCTATTTACTTTTGTTATTGAAATTCCCATGAAACGCTTTGCTTATTTCGATTTCAAT

**GGCGG21**

GCATTGTTGTTGTCAGTTGTTGTTTGGGGCATGTTTTGCCTCCTCACGCCGTCCAGCCACCCCCGCTACACCACCGTCTGACTCTTAGAGCTGTCAGTGAGCTATCCCAGCATCAATCATAAATTATAATTCACTGACTCACACAAT

**GGAGT21**

GGATTTAGATTCCGATTTTGCCATTGCTCTAGCGAAACAATGCACGATTTATTTATTTATTTATTTATTTATTTACAACTCCAGAGCATTGTGCTCGGTGCACTCGTCACATTGCGCTAGATTTGCTTGCCCTTTCTTTCTTCCTCTCTCCCTCTCCCTCTCTCTTGCTCTGCCTTTGTCTCTGTGTTCACT

**GCATT22**

GCCCATAAATGCGTCATTGGCAGTCGACGACTAAAAAGCAATTGCTTCATGCTCTGAAATATGTTTTATTGATTTCCTGTAAAGCTGTAAGAATTGTAAAATAAATGTGTCGAAAACGCGCTGCAATGGCAATAGTCAATAGCCATATGGGTAGCTGGCAGCTGGTAGCTGGCAGCTGGTAGCTGGTAGCTAAAGGGATCCCTTGGGCTGCTGACATTGTGCACACTTTTGCTGGCCATAAT

**GCAGT21**

ACCGAGTGAAATGCCAATGAGCTAATGGGCTTTCATTGATCTTTGGGCAGGCAATTCATGATTTCTAATCTCTGTCAATTTGTGAGCCGCATTAGAAAGAGCTGAGCCAGTTTTCAGGCAGCTTTCAAATAGAACCTTTGCTGTGTTTAGAAATTTGACTGTGACTTTATTTAGTTGCTACTTTACTCAGGACTCATCA

**CCCCC22**

CCATTTTCAATGGGTTCAGGGATTTTTTATTTGCACTTGTCGCTTGCCACTGCTCGCCGGTCTGTCTATATGCCTGCCTTACCGTCCTTCCGCTTGGTCACTCTAAAAATAGCTCAGCGGCACTTGCGACTTCACATTTGTTGTCGCACTCACGCTCCCTTTATTTGCCATTCTGTTGCTCTGCGTCGCTTGCTGGGGTATCCGTAGGTGCCCGTGTGAGCAAATACATCTGATTTTGGATGCTAAATGATAAACTGCCTCACTGCAAGCAGAGGACACAGTTTGGGTCTGTTGCGTCGACATTGTGCTCAATTGACAACGAGCCTCATTTTGATGCCGTGTGTGTGTAACATTTCGCATGTCGTCCTTGACCCAAAGCTGCGCCGGTAAATAAAGAATCGACGCCTTGCCTAACAGTTTCCACTCACACACATATACATAAACACACACATACATACATACTAATGTACATATAAATGTATTTATTACCAGCCTCACCACCCCAGGCAACGTAACTGGG

**CCCCC23**

CAAGAGCTTGGCCTCGACAACAGAGGCTGACTCGGCTAGTTGATCTAATTGCTGATTGCTGGCGATGTCGCAACTGGCCAAGGATAGCGTGAACAGTTGCTTCAGCTCCTCCACAAAGGCGGGTAAATTCGATTCGATACGTACGAGTAGCTCACGTTCGTAGCTCTTGAAGAACTCAACATACATCTTGCGTAGATTCTCCCAAC

**CGGCA22**

CGCCCTGCTGTACGACATGATTTATACGCAGCCAGTGGTATATGAATTTGGAAAAATTTCAGTGCCCACAACGGTGATGATTGGGGCGAAGGATAACACCGCCATAGGCAAGGCAGCAGCCCCGCCGGAGGTTCAGAAACAACTGGGCCATTATCCAGAACTGGGTAAAGCTGCGGCCAATGCGATCCCTCATGCGCGCCTCATTCTGTTTCCGGACCTTGGACACTCGCCTCAGATTCAGGACCCGGTTTCCTTTCATAAAGCGTTGCTGAAGACGCTTTCTCCCGAGCAATAGAGCGCGAATTGGAGAGGCGTATTTCGTCTTTTCATGCTGTCTTTGCAGATCATTTTTCAGGTGTGAAAGATGACTGCTCAGCGTGGGTGCTTAGGGCGCGTTGCCGTGTGGTGTGAGTGAGGGTTTTTTCTGAAATGGGTCCATGCTGTGCAGCAAAGGGCTGCCAGAGCAGCATAGATGC

**CGGAT22**

CGTACTGTCATATTGTGTGCAAATAAACTATAAATTTGGTTTTATAATCAACTGAAGTGCTTGAGCTATATAAATACAAAAATTGTTTGAGTAGTGTTTATAGCATTACTGTAGACATTGAGACTAGACGAGCCAGATGAATTTCAAAACACAATTTCACTGTAGTCCGCCCTGAAAGAGCAGAGATATTTTTTATTATGTAGCTATTATTATCAGTATATAATC

**CAGAT21**

CATAAAGATGCCTGGGGCGGTGAAAACTGCTTTGAGCATCTGAAAGAATACATCAGCAACCCGCAGGATAACCATGATGTACTTGAGTTTTACTATCTGATCCTGTCCATGGGATTTGAAGGGAAATACCGGATGCTTGATCGCGGGGCGGTATTGCTGATGGATCTGCGCAATCATCTCAGCGGCCTGCTTTATGCACGGGATACAACGCTGTATTTGTCCGATGTCCGGGCGGAGAAAGTCAGGCCGCCGAAACGCCGTTTTATCACCGCATTACGCATTCTCATCACCGGGCTTATCCTGGCGGTTATCGCCTACGGTATTTCAGCCTGGTATCTCCATGAGCAGGCGCGCCAGATACGCAATGATATTCTGGCATGGGTACCGCCGGAGCCGCGTAAAATCAATATTATGGAGACACTGCCTAATCCGCTGACCCAAATC

**TGGAT21**

TGCGATATAGCCGGTGCCGCCCGCGCTGCCGGTATGGCCGATAATCGCCAGGACAATATCTTTGGCTGTGATCCCGGGAGCAACGTTGCCGCGTACTTCAATTTTCAGGGTTTTGGCACGAGCCTGTTTCAGTGTCTGGGTGGCTAACACATGCTCGACTTCAGAAGTACCGATACCAAAGGCCAGTGAGCCGAATGCACCGTGAGTCGCCGTGTGCGAGTCACCGCAGACAATAGTCATGCCCGGCAGGGTGATGCCCTGTTCCGGCCCCATCACATGCACAATACCCTGGTACGGATGATTCAGATCATAGAGACGGACACCAAATTCCTCACAATTTTTGATCAGCGCCTGCATCTGGTTCCGGGCCATTTCACCGCAGGCATTGATGTCTTTGGTTTGCGTGGATACGTTGTGATCCATGGTGGCAAAGGTTTTCTCCGGGCGGCGCACAGGGCGGTTTTTCGCCCGTAATCCGTCAAACGCCTGCGGTGAGGTGACTTCATGAACCAGGTGGCGGTCGATATACAGGATCGGCGTTTCGTTTTCTTCTTCACGTACCACGTGGGCATCATATAATTTCTGATATAAGGTTTTTCCTGTGCTCATC

**TGTCG25**

AAAAATTTAAATGGGCTGGGAAACAGGGCGGAGCCATCTACCAGCCGCCAAGTGGGCCGCGGGCATCGATTGATCACATGAAGTTATGGTCGGGCAGCTTCGTGGATCTCCAAGCCTTTTGTTGCGTTCTCGATCCGTGGGTGATCGATGATGATACCTAACCTAGCCACGAATTATGGGAAGTTGACTCAACTTCCAAGATCTCGTGGCTGGTTGGGTCCCTGATACATCAGACGTGTCTCGACTGTTCGTAACAAAAATCTGTCGTTTCCTGGGCAGGCCCCCGCATAATCGTACCCAGTC

**TGTCG26**

TGTGGGGCAACGTTATAGGTTTCTTCCGCCGTAAAAACAGCTAACGCAGCATCCACGTCAGGTTTCACAACCCGAGCGACATCCGTAAGAGATCTAATAAGATCCAGAATTTGAGGCTTGAAGGTTTTCAGATTGTCGGTCAATTGTAATAACAGCTTCACCTTGCTGGCTGTTAGTTTGGGTGGAAAATCAACGA

**CGCCC21**

CGNGGCNCGNNCATGGGCGGGCCGCGGGAATTCGATTGACTGCGTACCAATTCCGGCGTTCCTGGCATCTGGTGCAGCCGCACCTTGCGATACCCCGGACGGCGCAGATACACCCGCACATTCCCGTGCCTGTCTGTGTCCTCTGACACGTATTTATAGCGCCGCTTCACCTGCATCACTTTCTTACAACCGCATCCCACTCATTTGGCGCATCCTGCATTTCCGTCTGTGCGGAATAATGCAGTTCAGTGCCTGTCATTTTTGCAGCCGCTGCCACCAAGGCTGGACGAAACCAGAGAGACAAAGAGCCTACGCGCGTAGGCCGTGGCACCAATCCCATTTCCACCATTTTATCAAAGGTGGTTTCGCTCACATCAAAGAATGCAGCCGCTGCCCGCCGTCTGAGGATAATCGGCAAAAGACCCGGTGGCAGATGGTCATATCTCGATTTCATGCCGCATCATCCTGCTTCGGG

**CAGCG22**

CGGGTAACGGTTGAGTTTCCAACCTGATTTATAAGCAGCAAGGGGAATATGTAATTATGAAAAATTGAGGTGCTGAAAACCTTGTATCTTGAAATTCTGGAAATTATTACATATGGAAGGGATGTTGCTCGGGTTGGGAGTCAGAGTAGAATGGTGCATCCAAAATTGGTAAAGGCTAAGACAGGTGCTTGACTGCAAGCTGTAATTGGTACGTGTTCGATC

**GGCTC23**

GGGCACGATTCCGAATCGTGACAAGAAGTTCCTCCAGAAACCCTTCCGAAGAAAACAGATCTGCGGTAGAAGGGTTTTCTTTCAGCATGTTCATTTTACCCGCCGATTTGCGAGGGCACGCATGTGTCCCCGTCGTTTCGATCAACACCCTCACCCAACCAACTCCGAAGCCTGTTCAAGGCGGACTCCCCAGCTGGCTCGACAAGATCAAAACTACCCCATATTGGTTTCCTTTTCAACTATTATGGGGTGGTTTTTTATTTCAGATAAAGTCCTCAGAAAGACACCCCACCCTGAAAGTACCACTGCCGGCCACGATCAAACGATTTCTGGTAGTTGCCGAAGCTACCTGAATAGCTACCCCACGCCATAAATTTCTCGTTTGTCGCGTTATCCAATCCAACCATAAAGTTATACCGCTCATTAGCGCTCATCCACCGGGGAG

**CAGCG24**

CAACGCCTATCTTGAAGGTTTTGGCGCCGGCTCCTACAGCCGCGTGGATGCCCAGTTCTATCAGGGCCTGAACCAGGGTGTTATCCGCAACACCGATCTACCGTTCGTGCTGCCCCGTTATACCTACAGTTTCCTGAGCCAGCCCGATGCATGGGGTGGCCGCTTTGGCATGTCCACCACGGACTTCAACGTGAAGCGTGACAACGGCACGTCGGACATCCGTGGCCAGATGGCGCTTACTCAGGACTCATCAGCCCTGACTGCGTACCAATTCCAAGCGACGCTGACAACGCCCCAGTACAAGGGCGAGCACACCTTCGGACTGAGCGGACACTATGACAGGCCCAGTGCGGATTATGTGAACTTTGTTTGTGCCCTGGATTATCCAGCCAGTCGTCGCCTAAGCGATTTGGATGTGACCTTCAGTTCGC

**CGGAT23**

CGAGTATAAAGTATGTGCCCACTGCAAATCAGCTCATAAATAATTTCGGGGGCCGCTGCAGTCGCCCTGCAGCCGAAAACAAAAGCAATGTGTACGAATTTTGCTCTCAAGTTCAGTGCGATATACAACAACGAGGGCGAGCGGTAGATAGCGAAAGATTCGGTATAACCGAGAGCTGTTTATGTTTATCCACTGTCAGCTGTCAACGCCAGGGCAAACATTTCCCGGCGATGTCGAGCAGCTGACAGTGGCTGTCAGCCCTCAACCCTCGTGACCGGGACATTGTATCTGGTGTATCCGTAGTTGGTGTGTCCTCTGATTGACGCCCATTACACCTCCCGCTCCACTCCCGCTGCTGCCTGACCTTGAATAAACAACAGACTCCGAGCAGCAGCAACATCAGAAACAACCAAAAATTTTCCGAATTACTTTTGAGGATC

**CGGCG22**

CGTTGGCCAAAAACCAAAAAGCCAGCCGTTAGCAGCATGTGCAGTCAACTGGCTGGCATTTCGGCAAATACATTTGTAATTCAAATGCAAATTTTTGGCAAAAGAAAAGTATTTTTTTTCTGTTCTTTTCGTTCGTGTGGCCGTAAATTGTGAAACAGCTGCGC

**CGCCC24**

CGGCTGTCATTTTGCTGGCGAACCGGAAGTCATTATCATGGCAAACAAAGCGCAACAGAGACGGTAGCCGCCACAGCTAACCACAGCTACAACAACACAAACACACCATTACATGGAGCATTTTGCGCCTCTGCGACAACTTGGACCGACTCCGGCTACGACTACGAGTTTGCCCTCATTATCGGG

**TGGGG21**

TGGCAAGGGCGGTACAGTGACATCCGCCATCTGGAACATTATCTGGCCCGGCAGGGAACCATCGTTCTAAAGTTCTTTCTGAATATTTCACGCGAAGAGCAAAGAGAACGCCTTCTGGCCCGACTGGATATTCCCGACAAACGTTGGAAATTCTCGCCTGCGGACCTGCGTGAACGCGAATATTGGGATGACTATCAGAAGGCTTATCAGAATGCCATTTCTGAAACTGCTCGCCCTTACGCGCCTTGGATTGTCGTCCCTTCCAATCACAAATGGTATGCACGTCTGGTAGTGATTGGAGCCATTATCCGTGCGCTGCAGAACCTGCAACAGCAAGCCCCCAAGCCTGCACCGGAAGTTGCTAAATTTCTGGATGATTACCGTACGCGCCCC

**TGGCG23**

TGGACTATCAGCATGCGCCTTGCAATACGCCCAGCCAGCCTCTTCAGCAACCAACTTCGACTTTTCATACAACGAAAGCCCCGGTTGGTCTTCGCGGGTCCAGTCCACTTCAGTGACGGGATGATCATGATCCAAACGACTGAAGCCAACGGCACCAAGGTTGGCAGTCATGACGACCCGC

**TGGCA22**

TGCACAGAGACAGAGAGAGAGAGAGAGAGAGAGAGAAGGAGCTGCATGGAAAGCGTCTTGAGTTGAGTTTCTTATTAGTCTGAACAAAAAACAAGACTGCAACCAAAATAATGCGGTCTACTGGCCAAAAATCAAAGCAAGCAACCAACAGACGAACTCACATACACACATGCAACATGCAAACATATTTGAGAGTATATCTAAGATGTTTGC

**TGGAT22**

TGCTTACTCAGTTCTTGAAATATTCTAATAGTTCTCAATTCGAAATCTTTACCACAGAGAAAAAATATTCGAAATCTTTACCACAGAAAAAAAAACCAGACTAAAGCTTTTATTTCGTAATTTATCGATAAAAGAGTATCAATGAATACTTTATTTTATTTCTATTCTGAAATAACTATTTTCATAGAAATCTGTGTGATCTTTTGCAGATTTTCTAAAATGTTCCATATTTTCGAAATTTATGTCACTTTCGTTTGGGGTTTGCGCTAGTTTCTTTTTTCAATC

**TGCGG22**

TGAGAGCAGGCCTGACTGGTGCCGGCAGCCCCAGCAACGGCCCCCGTCACAACAGACCCGTATTTTCCACACCCGGCGGACTGATTGGCAAGCTGCCAGGCGCCGACACCACTCGTCCCACCATTACTGGGGCGGACAAGCGCCACAGGCGTGGGTCCAAAGTTATTGATGTTGCCTGCGCCATCAGAAACGTTTGTGATGCCG

**CGCCC23**

CGGCGCATTGATCCCGTGCAGAACAGACAGACGCTGTGCCAGCATCCGGCTCTCTTTTTCCAGTGCTGAACGACTGATTTCCGGTGTGGCATTCAGCAGCGACAGGGTAATCGCATAACGCTGTAATGTTTCACGGATCCCGGCGGCCAGCAGTTGTAACGGACGGATACGGGCCGGG

**TTTCG21**

TTTGCTCTCTACACATGTAACCGGGGACGTCTGGACCCGCATATCGTCACTGCCTTTGACGCCATACTGGCCGGTATGCCCGTCGACCGGGATCCTACCGAAATCCTGCGCAAGATCGTGTTTTCGCAAGATTGGGGACCTGACGAACTTGCCTATCTCGCAACCCTCCCGTCCGAGTGCTATGATAAGATGCTGAAGGACTGGCAAGGCCACGACCTCAATAATGCAATCTATCTTGCGCTTGACTTCGGTCGATTAGATACAAGCGCTACTAACAAGATCGAATTTGCACGACAGATGGCAGCATCGCTGCAGCGTATAGCCGCAGAGAGCGGCCTCAATCGA

**GTGCC22**

GTGGGCTGCCTGAATCACACCATAAGGCCCCCCGCCATCTGTCATGATGTAGTCATTCAGCACAATCAGCCCCGTATGCGGC

**TGATT23**

GACTGCGTACCAATTCTGTTTGCAGGCGTCTGGGCTGCATTAGTGTAGCACGGCATTGCTGTGCGTTGACAAGTGTCGGCTTTAGCTAAATACCCTGTAATCGCTATGGCTGCTGGCTGGCAGGACTCCCTCAGCCCGAATTACGCATTATCTTGTCAGCTTTTGTGTAGATCTTGTAATTTGAGGTGCGTTGAGAATATAAGAATATTATTTATGTACTCTATATACTCTGTTATTTGAGTGATGGGTATATTGATGCGATGTGAGACTTGAAGATAAT

**TGATT28**

TGGGCTATGCCGGGCTAGGCTGGGCTGGCAATAAAAACGCACTTGGCTACCTCACTCGAGGCACAGCTCGTTGCCTTCAACAGTCTGTCGACTGGTTACCACTTCCGGCGGCAAAAGCGGTTCCTGCCACAGGTAACCAACGGCAACCCCAAAATGTCTGGCCTGAGTGCCAAGTCGAGTCGAGTCGACTCGAAGCGTTGCGCGTTCAGTTGAGCGTCTGGGAGCAACATATGCAAAT

**TTTCG21**

TTGAAGATCAATCCACAGCACACGATTCCCACTCTGGTGGACAATGGCTTCGCACTGTGGGAGTCGCGCGCCATTCTGGTCTACCTGGTGGAGAAGTACGGTAAGACCGACTCGCTCTACCCCAAGTGCCCCAAGAAGCGCGCCCTGATCAACCAGAGACTGTACTTCGACATGGGCACTCTCTACCAGAGCTTTGCCAACTACTACTATCCTCAGTTGTTCGCCAAGGCGCCCGCCGATCCCGAGGCCTTCAAGAAGATCGAAACTGCCTTCGATTTCCCGAATACTTTCCTGGAGGGTCAGACCTATGTTGCTGGTGACTCACTCACCGTCGCCGACATTGCCCTGTTGGCCACCGTGTCCACCTTCGAGGTGGCCAAATTCGACATCAGCAAGTATGAGAACGTGAACAAATGGTACGAGAACGCTAAGAAGGTGACTCCCGGCTGGGA

**TTTCG22**

TTCTCACTACGACATATCAAGATGGTCGTACGTAAGGAAGACTTTCCTATCGTTTGCTACTGCGCCCTAACGGGCGTCAAGATCATCGTTCGTGGCGCACCGGACCGCACTTTCCAATTCATGGTGTGTCTCAAGAAACTGCTGCCCGAACCAATGCATAATCTAATGCGCATTGATGCACAGCATCAGCACTCGATCAGCTCGGAGTACAAGATTATATCGGTGTCCAATGAGATTGCTGTGCCCATAGCCAGCAGTTGCGTGCTCCGCATTGATTTCCTGGAAAAGCACATCAATGGCAATGATATCGTCTCAGTCAAGTGGCCCGGGGAACTTCCTCGAAAATGTAACTTGAACTGTTTCGA

**TGGCC21**

TGTCCCCTCAGCAGAGCTGCAATTCCAATTGCGATTGCGACAGTCTAATATGCGTCATGCAAAGGAATATCCAACCATTTCCGAGCATTGCAAGCTCTGGCCAGCTCAATAGTGTGCAACACTTTATTCATCATTGAAATATATAAATATATATATAATATATGGC

**CAGAT21**

CAATAGGGTCAGACCCACGCCCTTTTCCACTTGGAACACAGCCTTCTCCGAGATGATCAAATCGACAACGCCCTTACCGGTCAACGGCAACGAGCAGCTCTCGAGAATCTTAGGCGAGCCATCTCGGGCATTGTGCTCCATGGTAATGATGACCTTGGTGCCTGGGGCCGCCACCAGATCCATGGCGCCGCCCATGCCCTTCACAAGCTTGCCCTGCATGAGAAAGATATTACATTAGTGTTTACTATATTACCATATTAGTTTGCATTTCTATTTTTCTTACGGGTATCATCCAGTTGGCCAAGTCGCCCGTTGCCGACACCTCCATGGCGCCCAGAATGGTCAGGTCTACGTGGCCATCACGAATCATGGCGAAGCTTTCATCGGAACCGAAGTAGGCAGCACCTGGCATAACCGTAACGCTCTCCTTGCCCGCATTGATCAAATCTGGATCGACCTGATCCTTGGTGGGGAATGGACCCAGTCCCAAGATGCCATTCTCCGACTGCAGCATGACGTTCATGCCCTGTGGTATATAGTTAGAGGAGAGCACTGGAATACCAATGCCCAGATTGGCATACATGCCATCCTG

**CAGAT22**

CAATAGCACGTAGGAAGCGGCTGTCGGTGTTGCCGGGACAGACGTACGTATGGTATTTGACGTCGCTGTTGGGTGCGAGAAATGTGAAGGAGGGAAGCAGATTGAACATTGACAAATACTACTTACAATTCCTTCAAGGCGCTCTCAAATGCTGTCCAGAATGGATTCGAGTCGTCTACCTTAGTGGGTGGCACGTATTCGTCTTTCTGCTCGAATATGATCTCAATGCCACCGCCGGCCTCCTCGCACCAGTCGCGTATCTGCTGCTCGAAGGCGACCAGATCAACATCGATGGCAATGCGCAGATCGAAGACCACCTCGATCATGGCGGAACCACGTTGCGTTGCACACCGCCCTCCACGACTGTCAGATTCACAGTGGTCACATCACCGTAGAAACGATTCTTATCGTCCTTCAGCGCTTGGACCTGGGACGCACGGAATCCCATCATCTTGCCCACAATATAGTTCAGCTTCTCGCCAGCTGTGTTGGGCAATAGTATGGAGCCATGGCCCGCAGTTCCGCTGATC

**TGTAC21**

TGCGATGTTCAGGCTCAACCCTGGCGCCAATTGGCCAAAGTTCTAAACAATGGCAGTTTTATTGGTA

**GGGCG21**

GGCGGCCTGTTGGGCGGCTCTGGTGGATGCTCGGCGGCTGGAGGTGCTGGTGGTGCGCCCAAGCTATTGGGCAGCTTTGTGATCAAGGAGAACTTTGATGTGCATCCCTCGCACAAGGTGGAAGAGGGCATCTTCCAGCATATGAACAAGCTGCCGTCGAAGAAGAAGGATACGCTTACTCAGGACTCATCAGCCCTGACTGCGTACCAATTCGGTGCACATCAACTTTACGCCCACGATTCCGCATTGCTCGATGGCGACGCTGATTGGGCTCTCCATACGGGTAAAGCTGCTGCGATCTCTGCCGCCACGC

**GGGCG22**

GGTGAAGCGATTGCGAATGCCCACTGGCAAGTCCTTTTTACCAATATCCGTATTAGGATTCATGCATGCAAATATACGAAAGTCGGGATGACGCTTACTCAGGACTCATCAGCCCTGACTGCGTACCAATTCGGACATGAAAAAGAACACGGTCGGCTCGCTGGAGAGTCCGCGCAAGGACTTTGGCAAACGTTCGATCAACTGCAAGTCGCTGCCACCGCCAGCCAGCGATGAAAGCGGCGATGTGCTAACCACTGTGGTGGTTTAGCTCCTTAGCGATCTCAGCCAACAGCCAACAGCTGATAGCTGCTGCCAGCCACATAGCACCTCCAGCCCGAGGCCCATTCCCGC

**CGGGG21**

CGGCGGAAGCGGGTGAGCCGCTCATGCTGCGCCGCATTGTCTCCCGGGAAGGCCGCTCACGCGCATGGATCAACGATCAGCCGGTGGGCATTACACTCATGCGCCGGGTGGCGGTATCTCTGGTGGAAATTCAGGGCCAGCATGAACAGATGGGACTGGCTGATTCCAGCACACATCGCGGCCTGCTGGACGCCTATGGTGTCCAGGCTTCTTTGCTGCGCGATGTTTCCACCACATTTGATAGCTGGCAGAGCCTGCGGCGGGATCTGGATACGGCCCGGAAAAACCTCCTTGAAGCCGCGCGGGAAGAAGAATGGCTCCGACATACGGTGGAAGAACTCACCGCCC

**GGGCC21**

GGCCGCAAGACCAAGATCGATCAAACCAGCTGCAACACCGACTATTCCTGTCTTGAAGGCGATTGCCCGTCATTCGTGACCGTGGAAGTGACCGACGACACCCCCCTGCCGAAGAGCCAGGATGCCCCGCGGCCACCTCACGTCGCAGATCCCAAAATTCCTTCATTGGGCGCTGATCCGCACAACATCCTCATGGCCGGGGTCGGTGGGACTGGAATCGTCACCGCCAACCAAGTGTTGGCAACAGCGGCTTTGCGGGCGGGACTTTACGTCGAAGGTCTTGATCAGACGGGC

**CACTC23**

CTCTCCCAAGCGTGTGCTATGTTTTTGATATCCTACGAGTCTAAACTAGCCAGCTCTCGCCACTGGAAGACACAGGGCTTTTATCTATAGTAGGCGCTTCGACCAATGGCCTCATCAAAAGACAGAGGGACTTGGATGGCAATTTATTGTCCACATGTTTCACGTACAACATTGTAAACGAGGGAAATATCATTGCAACAACGTGTCCTTCCCAAATTAGGAAGGCTTTCTTGCCAAAACAGACCCAAGCCTCATATCTGGAACCACCCCTTCGTTGAATGAGCTTTCAATGTCGCCGTCGAGTTGATGAAGCTGTGATTCGATTTCTTCCTTTGAGTGTCCTTGTCTTTTCCATGCAACAGGCAATAGCGCTTTCGTGGTGGATTTGAACCAATCCTGGACCAAAGGATTGAGTTCTGGCTTAGATAGGTTGGTGTACTCCATAGAAACAATATCAGACAGGCCTTGGGTTTCATATTCCTCTTCAATCAAAGTAGGGAGACGACCGCTGAAGCCCGCCTGCGCGGCGTAGCGATAGAGTGTTGTTGTATATGCCTCTAAGGTTGCATCTGGATCGGAGTACCGCATGCGCTCCGTGTG
